# Dung Beetle with Reflection Cuckoo Catfish Optimizer for Numerical Optimization and Reservoir Production Optimization

**DOI:** 10.3390/biomimetics11050306

**Published:** 2026-04-30

**Authors:** Shengnan Li, Taiju Yin

**Affiliations:** School of Earth Sciences, Yangtze University, Jingzhou 434023, China; lisn.stu@yangtzeu.edu.cn

**Keywords:** Cuckoo Catfish Optimizer, adaptive local search, reflecting boundary mechanism, bio-inspired algorithm, reservoir production optimization

## Abstract

As engineering systems grow in complexity, reliable metaheuristic optimizers are increasingly essential. While swarm intelligence algorithms are widely applied, recent approaches like the Cuckoo Catfish Optimizer (CCO) can experience premature convergence due to limited local exploitation and simplistic boundary handling. To address these limitations, this paper proposes the Dung Beetle with Reflection CCO (DBRCCO), integrating two principal mechanisms. First, an adaptive local search strategy inspired by dung beetle foraging is incorporated to intensify exploitation within dynamically contracting regions. Second, a momentum-preserving reflecting boundary mechanism replaces traditional clamping, maintaining population diversity near constraint edges. DBRCCO is evaluated against eight contemporary metaheuristic algorithms using the 29 CEC2017 benchmark functions and a reservoir production optimization problem. Statistical analyses indicate that DBRCCO achieves competitive performance, securing a Friedman ranking of 1.5172 (p<0.05). In the reservoir application, DBRCCO improves the mean Net Present Value (NPV) by 12.54% while reducing variance by over 72% relative to the standard CCO. These findings suggest that DBRCCO offers a stable and effective alternative for complex optimization tasks.

## 1. Introduction

Optimization is critical across domains such as engineering design [1,2,3], economic planning [4,5], and scientific discovery [6]. However, real-world problems often involve high-dimensional search spaces, nonlinear interactions, and multimodal fitness landscapes. Classical techniques, like gradient descent [7,8] and the simplex method [9], typically ensure exact convergence in convex environments but often struggle in nonconvex and high-dimensional spaces. They are susceptible to local optima entrapment and the “curse of dimensionality” [10], which dramatically increases computational costs [11]. These limitations highlight the need for more robust and adaptive optimization frameworks [12,13].

To address these challenges, metaheuristic algorithms have emerged as powerful alternatives for resolving complex numerical problems [14,15]. Unlike traditional methods that rely heavily on explicit gradient information, metaheuristic algorithms operate on higher-level adaptive strategies that remain largely independent of specific problem architectures [16]. This independence enables robust search performance across deceptive fitness landscapes [17]. The core strength of these algorithms lies in their global search capabilities, which mitigate the risk of premature convergence by exploring vast, nonconvex spaces. By using stochastic sampling, population-based evolution, and adaptive learning mechanics, these algorithms provide a flexible framework for tackling high-dimensional and discrete optimization tasks where classical approaches fail [18]. This intrinsic adaptability positions metaheuristic algorithms as an active area of research in modern computational operations [19,20,21].

The taxonomy of metaheuristic algorithms is commonly organized into two principal categories based on their natural inspirations: Evolutionary Algorithms (EAs) and Swarm Intelligence (SI) algorithms. EAs are grounded in the principles of biological evolution [22,23], employing a population-based approach that iteratively refines solutions through simulated natural selection, crossover, and genetic mutation. Prominent examples include the Genetic Algorithm (GA) [24,25] and Differential Evolution (DE) [26,27]. Conversely, SI algorithms mathematically model the collective, decentralized behavior observed in biological colonies [28]. These SI algorithms achieve global optimization through the dynamic interaction and cooperation of multiple independent agents following localized behavioral rules [29]. For example, Particle Swarm Optimization (PSO) emulates the social dynamics of bird flocking [30,31], and Ant Colony Optimization (ACO) abstracts the pheromone-based foraging behavior of ants to resolve discrete combinatorial pathfinding problems [32]. Recently, the computational landscape has been expanded by novel optimizers inspired by diverse natural and social phenomena, such as the Slime Mould Algorithm (SMA) [33], Hunger Games Search (HGS) [34], Parrot Optimizer (PO) [35], Educational Competition Optimizer (ECO) [19], Moss Growth Optimization (MGO) [36], and the Beaver Behavior Optimizer (BBO) [37]. These optimizers introduce diverse search mechanisms that expand the versatility of modern evolutionary computation.

Despite their widespread adoption, the performance of metaheuristic algorithms is bounded by the No Free Lunch (NFL) theorem [38]. This theorem states that, when averaged across all possible optimization problems, no single algorithm can consistently demonstrate universal superiority. Consequently, although general-purpose EA and SI methods offer robust baseline capabilities, they may still exhibit suboptimal convergence behavior [39], computational inefficiency, or limited scalability when applied to problems featuring restrictive boundaries, extreme dimensionality, or complex coupled variables [37,40]. This limitation underscores the need for algorithmic refinement tailored to specific application domains and equipped with adaptive control mechanisms [41].

This study focuses on the recent Cuckoo Catfish Optimizer (CCO) [42], an SI metaheuristic inspired by the brood parasitism of *Synodontis multipunctatus*. CCO balances exploration and exploitation through simulated spatial compression and chaotic predation. However, empirical observations indicate that its reliance on broad stochastic jumps can present structural limitations. Specifically, the absence of a fine-grained local exploitation mechanism near promising optima may hinder convergence precision. Furthermore, its basic boundary-handling approach—which typically re-initializes out-of-bounds agents—can discard search momentum and contribute to population stagnation near constraint limits.

To address these challenges, this paper proposes the Dung Beetle with Reflection Cuckoo Catfish Optimizer (DBRCCO). DBRCCO introduces two targeted modifications to the baseline CCO. First, an exploitation operator inspired by dung beetle foraging is incorporated to facilitate structured local searches within adaptively contracting regions around historical best solutions. Second, a momentum-preserving reflecting boundary strategy is applied to retain kinetic search behavior at spatial limits, helping to sustain population diversity. These additions aim to complement the global exploration capabilities of the original CCO, thereby supporting improved solution accuracy.

Rather than sequentially concatenating independent algorithms [43], DBRCCO embeds the Dung Beetle Optimizer (DBO) exploitation behavior as a conditional local search phase within the standard CCO lifecycle [16,44,45]. When agents are identified as stagnant or operating in high-potential regions, the algorithm temporarily shifts from CCO’s broad exploration to DBO’s localized foraging rules. This conditional coupling allows the framework to dynamically respond to the fitness landscape, focusing computational effort where fine-grained exploitation is most needed. While invoking this secondary local search operator introduces a marginal per-iteration computational overhead, empirical tests suggest it facilitates earlier convergence, providing a favorable trade-off between iteration complexity and overall search efficiency.

The remainder of this paper is organized as follows. Section 2 briefly reviews the original CCO algorithm and its mathematical components. Section 3 details the proposed DBRCCO framework. Section 4 presents the experimental results on the CEC 2017 benchmark suite and corresponding nonparametric statistical evaluations. Section 5 evaluates the applicability of the algorithms on a synthetic reservoir production case study. Finally, Section 6 concludes the study and suggests directions for future research.

## 2. Original Cuckoo Catfish Optimizer

The Cuckoo Catfish Optimizer (CCO), introduced in 2025 by Wang et al. [42], is a population-based metaheuristic inspired by the obligate brood parasitism uniquely characteristic of the *Synodontis multipunctatus* (cuckoo catfish) native to Lake Tanganyika. The algorithmic model simulates an artificial school of *N* catfish agents systematically navigating a *D*-dimensional search space to locate an optimal host nest, which represents the global optimum. Its core mathematical framework emulates the biological sequence of host location through four primary stages: cooperatively compressing the prey’s escape space, executing a multidirectional surround search to encircle the target, introducing chaotic stochastic disturbances during the final predation phase, and applying a parasitic replacement strategy to evaluate and replace underperforming individuals. This structured workflow is designed to maintain a robust theoretical equilibrium between global exploration and local exploitation.

### 2.1. Mathematical Model of CCO

The mathematical model of the CCO framework translates biological behaviors into computational search operators for numerical optimization.

*Notation Statement*: Throughout this paper, operations involving the addition or subtraction between a scalar and a vector are defined as the scalar being applied element-wise across all dimensions. Additionally, the symbol ∘ denotes the Hadamard (element-wise) product between two vectors.

**1. Initial Population Generation:** CCO initializes the search process by uniformly distributing a randomized population of *N* agents across the feasible search space. The initial position of the *i*-th agent in the *d*-th dimension is defined as

(1) $$\begin{array}{c} x_{i}^{d}=r_{0}\cdot \left(u^{d}-l^{d}\right)+l^{d},\;i=1,2,\ldots ,N;\;d=1,2,\ldots ,D\; \end{array}$$

where $x_{i}^{d}$ is the *d*-th dimension of the position vector $x_{i}$. The parameters $u^{d}$ and $l^{d}$ represent the upper and lower bounds for the *d*-th dimension. The parameter $r_{0}$ is a uniform random scalar in [0,1].

**2. Compressed Space Strategy:** This strategy models the cooperative behavior of catfish compressing a target search area, effectively restricting the prey’s escape space. During early iterations, an individual agent $x_{i}$ updates its position vector by integrating spatial information from randomly selected peer agents and the current global best solution $x_{best}$. This mechanism uses intra-population positional relationships to focus the search. A self-protection safeguard is also integrated, preserving trajectory momentum when the population fails to discover superior solutions, which helps mitigate premature convergence. The position update equations are defined as

(2) $$\begin{array}{cc} x_{i}^{\mathrm{new}} & =x_{i}+Z_{1}\cdot \left|r_{n}\right|\cdot \left(\frac{x_{best}+x_{r1}}{2}-x_{r2}\right)+\frac{r_{1}}{2}\cdot \left(x_{r3}-x_{r4}\right) \end{array}$$

(3) $$\begin{array}{cc} x_{i}^{\mathrm{new}} & =Z_{2}\cdot \left(x_{r1}+\left|r_{n}\right|\cdot \left(x_{r1}-x_{r2}\right)\right)+\left(1-Z_{2}\right)\cdot x_{i}\; \end{array}$$

where $x_{r1},x_{r2},x_{r3},\;\mathrm{and}\;x_{r4}$ are distinct, randomly selected agent vectors; $r_{n}$ is a scalar drawn from a standard normal distribution; $r_{1}$ is a uniform random scalar in [0,1]; and $Z_{1}\;\mathrm{and}\;Z_{2}$ are binary random scalars designed to simulate environmental search resistance (taking values of 0 or 1).

**3. Surround Search Strategy:** This phase models catfish encircling prey using spiral or spherical trajectories, facilitating exploration of the search space. The strategy encompasses two sub-mechanisms, executed with equal probability:

- **Spiral Encirclement:** Agents move along a logarithmic spiral trajectory directed either towards or away from a randomly chosen peer agent $x_{r}$, depending on a comparison of their respective fitness values. The position update is governed by
  (4) $$\begin{array}{cc} x_{i}^{\mathrm{new}} & =x_{e}+F\cdot r_{1}\circ \frac{s_{\mathrm{step}}}{2}+T^{y}\cdot s_{c}\cdot \left(1-r_{1}\right)\circ \left|s_{\mathrm{step}}\right|+V\cdot \frac{J_{i}}{t}\; \end{array}$$
  where $x_{e}$ is the target tracking vector (either $x_{i}$ or $x_{r}$); F∈{−1,1} is a binary direction factor; $r_{1}$ is a uniformly distributed *D*-dimensional random vector in ${\left[0,1\right]}^{D}$; $s_{\mathrm{step}}$ is the dynamically calculated attraction vector; *T* is a nonlinear shrinking scalar; *y* and $s_{c}$ are fixed logarithmic spiral geometric coefficients; *V* is an adaptive velocity scalar; $J_{i}$ measures the aggregation density scalar of the *i*-th agent; and *t* is the current iteration.
- **Spherical Encirclement:** Agents execute a spherical search around a dynamically selected center (typically chosen from the current elite agents or the population centroid) within an adaptively contracting search radius *w*. The position update is
  (5) $$\begin{array}{cc} x_{i}^{\mathrm{new}} & =x_{\mathrm{rot}}+2\cdot w\cdot F\cdot \cos\left(r_{\theta 1}\right)\cdot \sin\left(r_{\theta 2}\right)\cdot \left(x_{\mathrm{rot}}-x_{i}\right)\; \end{array}$$
  where $x_{\mathrm{rot}}$ is the target center vector, and $r_{\theta 1}$ and $r_{\theta 2}$ are independent random spherical rotation angles.

**4. Transition Strategy:** To ensure a transition from global exploration to local exploitation, the population is partitioned into two equal components. The first half converges towards the global best $x_{best}$, whereas the second half diverges to explore the search space. The update mechanism is defined as

(6) $$\begin{array}{cc} x_{i}^{\mathrm{new}} & =\left\{\begin{array}{cc} \frac{C}{t}\cdot \left(r_{2}\cdot x_{best}-r_{3}\cdot x_{i}\right)+T^{2}\cdot l\left(D\right)\circ \left|s_{\mathrm{step}2}\right|, & \mathrm{if}\;i\;(\mathrm{mod}\;2)=0\\ x_{best}+x_{r2}+De\cdot \left(2\cdot r_{1}\circ s_{\mathrm{step}2}-\frac{r_{2}}{2}\circ \left(De\cdot r_{3}-1\right)\right), & \mathrm{otherwise}\; \end{array}\right. \end{array}$$

where *C* is a monotonically decreasing scalar, De=C·F is a directional scalar, $r_{2},r_{3}$ are random scalars in [0,1], $r_{2},r_{3}$ are random vectors in ${\left[0,1\right]}^{D}$, and l(D) denotes a *D*-dimensional Lévy flight step vector.

**5. Chaotic Predation Strategy:** This mechanism models the final invasion of the host nest, simulating the evasion movements of the prey. The intensity of this disturbance correlates with the agent’s proximity to the global best $x_{best}$, regularized through a vigilance factor $L_{x}$. The position update is

(7) $$\begin{array}{cc} x_{i}^{\mathrm{new}} & =\left\{\begin{array}{cc} x_{best}+F\cdot S_{0}\cdot \left(x_{best}-x_{i}\right), & \mathrm{if}\;J_{i}>J_{in}\\ x_{best}\cdot \left(1+T^{5}\cdot C_{y}\cdot r_{4}\right)+F\cdot S_{0}\cdot \left(x_{best}-x_{i}\right), & \mathrm{elseif}\;J_{i}>J_{in}\cdot L_{x}\\ x_{best}\cdot \left(1+T^{5}\cdot G_{s}\right)+F\cdot S_{0}\cdot \left(x_{best}-x_{i}\right), & \mathrm{otherwise} \end{array}\right. \end{array}$$

Here, $S_{0}$ is a step scalar; $C_{y}$ and $G_{s}$ are chaotic coefficients simulating small-scale and large-scale prey evasion movements, respectively; $r_{4}$ is a uniform random scalar in [0,1]; and $J_{in}$ defines the initial average aggregation degree of the population.

**6. Death and Parasitic Strategy:** This models natural selection. Stagnating agents that fail to improve fitness over consecutive iterations face a linearly decreasing mortality probability, denoted as $P_{die}$. Upon elimination, an agent is regenerated either near the global best or randomly within the search space, depending on the iteration stage. The regeneration rule is

(8) $$\begin{array}{cc} x_{i}^{\mathrm{new}} & =\left\{\begin{array}{cc} r_{5}\cdot \left(u_{c}-l_{c}\right)+l_{c}, & \mathrm{if}\;r_{6}>C\\ r_{5}\cdot \left(u-l\right)+l, & \mathrm{otherwise}\; \end{array}\right. \end{array}$$

where $r_{5},r_{6}$ are uniform random scalars in [0,1], and $u_{c}$ and $l_{c}$ define localized bounding vectors around the global best $x_{best}$.

### 2.2. Integrated Iteration

The CCO algorithm initiates a population of *N* agents within a *D*-dimensional bounded search space. During the iterative sequence, agents primarily execute the Compressed Space Strategy and Surround Search Strategy to explore the search space. A Transition Strategy then facilitates a shift from global exploration to local exploitation. In advanced stages, the Chaotic Predation Strategy is invoked to introduce localized searches near the best solution. Concurrently, the Death and Parasitic Strategy is applied to stagnant agents, regenerating individuals to maintain population diversity. The iterative cycle continues until a termination criterion is met, outputting the global best solution $x_{best}$.

### 2.3. Pseudocode of the Original CCO

The procedural steps of the CCO framework are summarized in Algorithm 1.
**Algorithm 1** Original Cuckoo Catfish Optimizer (CCO)  1:**Input:** Population size *N*, maximum iteration $T_{max}$ and problem dimension *D*  2:**Output:** The global best position vector $x_{best}$ and its fitness value $F_{best}$  3:Initialize the random population and calculate the fitness values  4:Set $x_{best}$ as the best position and $F_{best}$ as the best fitness of Cuckoo Catfish  5:**for** t=1 to $T_{max}$ **do**  6:   **if** t<15 **then**  7:         $P_{die}=0.02\cdot T_{max}$  8:   **else**  9:         $P_{die}=0.02$, C=0.810:   **end if**11:   **for** i=1 to *N* **do**12:         **if** $r_{a}>C$ **then**13:              **if** $r_{b}>C$ **then**14:                 Update the position vector $x_{i}^{\mathrm{new}}$ using Chaotic Predation Strategy15:           **else if** $r_{c}>C$ **then**16:                 Update the position vector $x_{i}^{\mathrm{new}}$ using Transition Strategy17:           **else if** $r_{d}>0.5$ **then**18:                 Update the position vector $x_{i}^{\mathrm{new}}$ using Spiral Surround Search and set $s_{1}=s_{1}+1$19:           **else**20:                 Update the position vector $x_{i}^{\mathrm{new}}$ using Spherical Surround Search and set z=z+121:                 Extract an integer *Q* in [1,4]22:           **end if**23:           **if** $s_{1}>10$ **then**24:                 Update the position vector $x_{i}^{\mathrm{new}}$ by jumping away and set $s_{1}=0$25:           **end if**26:           **if** z>5 **then**27:                 Update the position vector $x_{i}^{\mathrm{new}}$ by utilizing optimal position and set z=028:           **end if**29:         **else**30:           Update the position vector $x_{i}^{\mathrm{new}}$ using Compressed Space Strategy31:         **end if**32:         Update the position vector $x_{i}^{\mathrm{new}}$ using Death and Parasitic Strategy33:         Update the position using $x_{i}^{\mathrm{new}}$34:         **if** $F_{i}^{new}<F_{i}$ **and** Q≠1 **then**35:           $x_{\mathrm{worst}}=x_{i}^{\mathrm{new}}$36:         **end if**37:    **end for**38:**end for**39:**return** the global best position $x_{best}$ and the best fitness $F_{best}$

## 3. The Proposed Dung Beetle with Reflection Cuckoo Catfish Optimizer (DBRCCO)

This section details the proposed Dung Beetle with Reflection Cuckoo Catfish Optimizer (DBRCCO). By mathematically modeling perfectly elastic geometric reflections for boundary handling and introducing dynamic scalar bounds around the optimum, DBRCCO represents a hybrid advancement offering both theoretical and empirical insights. To address the original CCO’s vulnerability to insufficient local exploitation and inefficient boundary handling, DBRCCO integrates a localized search operator inspired by the dung beetle and an advanced reflecting boundary constraint strategy.

### 3.1. Dung Beetle Local Search Mechanism

This algorithmic enhancement introduces an exploitation mechanism inspired by the Dung Beetle Optimizer (DBO). It mitigates CCO’s reliance on global maneuvers by providing fine-grained refinement within promising regions.

The local search strategy comprises a Dynamic Spawning Region and a Dynamic Foraging Region, which adaptively contract as the search progresses. A dynamic contraction factor *R* reduces the search scope:

(9) $$R=1-\frac{t}{T_{max}}\;$$

where *t* is the current iteration and $T_{max}$ is the maximum number of iterations.

The Dynamic Spawning Region is established around the current local optimum candidate $x^{*}$. Its adaptive boundaries are confined within the global bounds [l,u]:

(10) $$l^{*}=\max\left(x^{*}\cdot \left(1-R\right),l\right),\;u^{*}=\min\left(x^{*}\cdot \left(1+R\right),u\right)\;$$

Within this localized region, the position of a simulated brood ball (candidate $B_{i}$) is updated using attraction and repulsion terms relative to the adaptive boundaries:

(11) $$B_{i}\left(t+1\right)=x^{*}+b_{1}\circ \left(B_{i}\left(t\right)-l^{*}\right)+b_{2}\circ \left(B_{i}\left(t\right)-u^{*}\right)\;$$

where $b_{1}$ and $b_{2}$ are random vectors with uniform components in [0,1], and ∘ denotes the Hadamard (element-wise) product.

The Dynamic Foraging Region is structured around the global best solution $x_{best}$. Its boundaries are similarly defined:

(12) $$l_{best}=\max\left(x_{best}\cdot \left(1-R\right),l\right),\;u_{best}=\min\left(x_{best}\cdot \left(1+R\right),u\right)\;$$

The position of a juvenile dung beetle agent (candidate $x_{i}$) is updated within this foraging region:

(13) $$x_{i}\left(t+1\right)=x_{i}\left(t\right)+c_{1}\circ \left(x_{i}\left(t\right)-l_{best}\right)+c_{2}\circ \left(x_{i}\left(t\right)-u_{best}\right)\;$$

where $c_{1}$ is a random vector following a standard Gaussian distribution, and $c_{2}$ is a uniformly distributed random vector. The assignment of these distributions is intentional. The Gaussian distribution governing $c_{1}$ concentrates its probability mass around the mean, inducing micro-sampling adjacent to the promising solution for exploitation. Conversely, the uniform random components ($c_{2}$, $b_{1}$, and $b_{2}$) introduce unweighted exploration. Their uniformity facilitates unbiased boundary testing, preventing spatial entrapment within local basins. This combination balances conservative refinement against divergent jumps.

### 3.2. Reflecting Boundary Handling Strategy

Another enhancement is the reflecting boundary control mechanism. This strategy sustains search diversity when agents encounter spatial limits, replacing the simplistic boundary clamping approach that causes population stagnation.

The reflection mechanism operates per dimension. When an active vector’s *j*-th component $x_{i}^{j}$ violates the boundary, it is symmetrically reflected back into the feasible domain. The reflected component ${\left(x_{i}^{j}\right)}^{ref}$ is determined by

(14) $${\left(x_{i}^{j}\right)}^{ref}=\left\{\begin{array}{cc} l^{j}+\left(l^{j}-x_{i}^{j}\right), & \mathrm{if}\;x_{i}^{j}<l^{j}\\ u^{j}-\left(x_{i}^{j}-u^{j}\right), & \mathrm{if}\;x_{i}^{j}>u^{j}\\ x_{i}^{j}, & \mathrm{otherwise} \end{array}\right.\;$$

where $l^{j}$ and $u^{j}$ define the lower and upper constraints for the *j*-th dimension. This operation preserves trajectory momentum and facilitates robust exploitation near the limits.

### 3.3. Implementation Framework of DBRCCO

**Figure 1:**
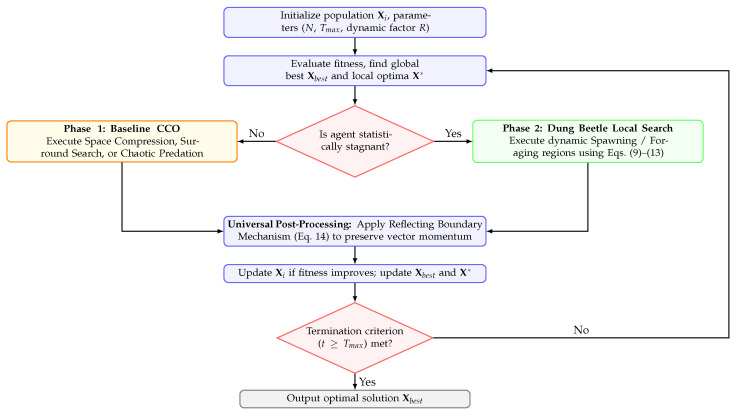
Detailed architectural flowchart of the proposed DBRCCO algorithm, illustrating the conditional integration of the Dung Beetle local search and the universal reflecting boundary post-processing.

The proposed DBRCCO architecture integrates these strategies into the CCO workflow as conditionally triggered operators, as illustrated in Figure 1. Non-stagnant agents execute the original CCO search phases. Conversely, agents identified as stagnant or residing within high-potential regions are subjected to the Dung Beetle Local Search Mechanism. This mechanism is invoked as a supplementary exploitation phase centered on either the global best $x_{best}$ or a local best $x^{*}$. The Reflecting Boundary Handling Strategy is applied as a post-processing step following every position update to ensure all candidates remain within the feasible space while preserving momentum. The steps are summarized in Algorithm 2.
**Algorithm 2** Dung Beetle with Reflection Cuckoo Catfish Optimizer (DBRCCO)  1:**Input:** Population size *N*, maximum iteration $T_{max}$, problem dimension *D*  2:**Output:** The global best position vector $x_{best}$ and its fitness value  3:Initialize the random population within bounds [l,u] and calculate initial fitness values  4:Set the global best $x_{best}$ and local optimums $x^{*}$  5:**for** t=1 to $T_{max}$ **do**  6:   Calculate the Dynamic Contraction Factor $R=1-t/T_{max}$  7:   **Population Status Evaluation:** Rank individuals and identify stagnant/elite ones  8:   **for** i=1 to *N* **do**  9:      **if** individual *i* is a **Standard Individual** (non-stagnant) **then**10:           **Phase 1: Conditional Baseline CCO Operations**11:           Execute Space Compression (intra-population relationships)12:           Execute Surround Search (spiral/spherical patterns)13:           Execute Chaotic Predation (disturbance near best solution)14:       **else if** individual *i* is a **Stagnant/Elite Individual then**15:           **Phase 2: Conditional Dung Beetle Local Search**16:           Calculate dynamic boundaries $\left[l^{*},u^{*}\right]$ and $\left[l_{best},u_{best}\right]$ scaled by *R*17:           Execute Dynamic Spawning Region update around local optimum $x^{*}$18:           Execute Dynamic Foraging Region update around global best $x_{best}$19:       **end if**20:       **Universal Post-Processing: Reflecting Boundary Handling Strategy (RBHS)**21:       **for** j=1 to *D* **do**22:          **if** candidate position vector $x_{i}$’s *j*-th component $x_{i}^{j}$ exceeds bounds $\left[l^{j},u^{j}\right]$ **then**23:                Apply per-dimension momentum-preserving reflection using Equation (14)24:          **end if**25:        **end for**26:        Evaluate the fitness of the new candidate position vector $x_{i}$27:        Update the individual’s position if the new fitness is better28:    **end for**29:    **Iteration Update:** Update local optimum vectors $x^{*}$ and global best $x_{best}$30:**end for**31:**return** the global best position $x_{best}$ and its fitness

### 3.4. Complexity Analysis

The computational complexity of the baseline CCO algorithm is determined by its population size *N*, problem dimensionality *D*, and maximum iterations $T_{max}$, resulting in a time complexity of $O(T_{max}\cdot N\cdot D)$. The integration of the Dung Beetle Local Search Mechanism introduces a localized cost of O(S·D) per iteration, where *S* is the dynamically selected subset of the population designated for enhanced exploitation. Furthermore, the Reflecting Boundary Handling Strategy incurs an overhead of O(N·D) per iteration for evaluating out-of-bounds scalar positions. Consequently, the asymptotic time complexity of the proposed DBRCCO remains $O(T_{max}\cdot N\cdot D)$, as the introduced operations do not alter the established order of magnitude. The space complexity similarly remains unchanged at O(N·D), necessitated solely for storing population vectors and their associated fitness values.

## 4. Experimental Results and Discussion

This section presents an empirical evaluation of the proposed DBRCCO framework. The objective is to assess its optimization performance relative to established metaheuristic algorithms across a standardized benchmark suite. All experiments were conducted under identical environmental conditions. Initialization parameters, including population size (*N*), problem dimensionality (*D*), maximum function evaluations (MaxFEs), and the number of independent executions, were synchronized across all evaluated optimizers. The primary performance metrics are the mean (Avg) and standard deviation (Std) of the best-found fitness values.

### 4.1. Implementation Environment and Parameter Initialization

All computational evaluations were conducted on a high-performance computing node equipped with an Intel Xeon Gold 6230 CPU @ 2.10 GHz and 64 GB of RAM running Ubuntu 20.04 LTS. The implementations were executed in Python 3.9 using the NumPy library.

The nine comparative metaheuristic algorithms were subjected to identical test constraints. Operating parameters across all optimizers were set to a population size (N=30) and maximum number of function evaluations (MaxFEs) of 10,000 × D. For DBRCCO, no supplementary hyperparameter tuning was required.

### 4.2. Benchmark Functions Overview

The performance evaluation utilizes 29 benchmark functions from the CEC2017 test suite, as summarized in Table 1. This suite encompasses four categories: unimodal functions (F1–F3), simple multimodal functions (F4–F10), hybrid functions (F11–F20), and composition functions (F21–F29). Unimodal functions evaluate exploitation capability and convergence precision toward a single global optimum. Multimodal functions assess the algorithm’s capacity to evade stagnation and explore the spatial domain. The hybrid and composition functions merge properties of different function types, generating irregular topographies that simulate complex optimization challenges. This diversity ensures a comprehensive assessment of the optimizer’s performance.

### 4.3. Performance Comparison with Other Algorithms

To evaluate the relative superiority of DBRCCO, its performance was statistically benchmarked against the foundational CCO approach [42] and seven contemporary metaheuristic algorithms: Hunger Games Search (HGS) [34], Parrot Optimizer (PO) [35], Moss Growth Optimization (MGO) [36], Particle Swarm Optimization (PSO) [30], Differential Evolution (DE) [26], Slime Mould Algorithm (SMA) [33], and Bat Algorithm (BA) [46]. These comparator algorithms were selected to provide a comprehensive evaluation. PSO, DE, and BA serve as classic evolutionary baselines, whereas HGS, PO, MGO, and SMA represent recently introduced paradigms. This balance ensures that DBRCCO is evaluated against both historical mainstays and current swarm intelligence methods. The comparative quantitative results, detailing the mean objective value (Avg) and standard deviation (Std) for every benchmark function, are presented in Table 2.

**Figure 2:**
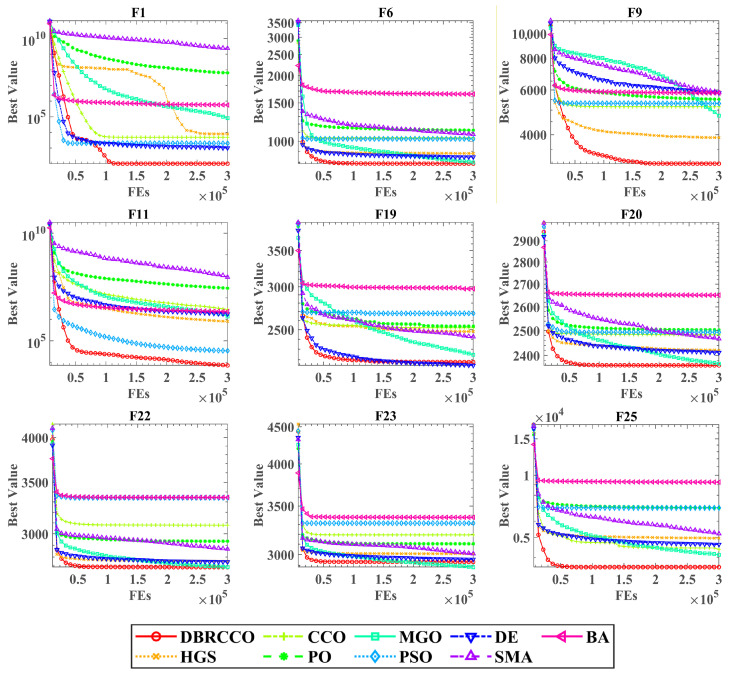
Convergence behavior analysis on nine representative benchmark functions.

The results in Table 2 demonstrate that DBRCCO secures competitive mean objective values across the CEC2017 suite. In the unimodal and simple multimodal categories, DBRCCO yields high convergence precision, frequently isolating the global minimum or discovering solutions superior to those of compared methods. Furthermore, its low standard deviation values confirm algorithmic reliability across independent executions. This stability validates the reflecting boundary handling strategy, which preserves search trajectory momentum and prevents population diversity loss when agents encounter geometric constraints. The convergence profiles in Figure 2 confirm that DBRCCO exhibits a rapid initial descent phase succeeded by localized refinement.

For the hybrid and composition functions, which feature dynamic scaling, rotation, shifting, and multiple local optima, DBRCCO maintains an advantage. It reliably isolates optimal configurations within most of these landscapes, outperforming modern optimizers such as DE and MGO. This performance highlights the contribution of the dung beetle local search mechanism. By adaptively transitioning between dynamic spawning and foraging regions, DBRCCO evades local traps while maintaining computational focus on promising sub-regions.

While dominant across most topologies, DBRCCO exhibited underperformance on specific functions, notably F3, F16, and F17. For function F3, the statistical disparity between DBRCCO and the leading algorithm is marginal. This lag is attributable to the precision of the Gaussian micro-sampling mechanism within the Dung Beetle local operator. While effective for exploitation, its focus can exhibit delays when tracking topological shifts intrinsic to F3’s dispersed landscape. For the composition topologies of F16 and F17, the underperformance is rooted in the elastic nature of the Reflecting Boundary Handling Strategy. These functions integrate asymmetric penalty ridges near their constraint limits. In such asymmetric topographies, a momentum-preserving reflection occasionally over-projects search agents past narrow convergence valleys. Recognizing this trade-off between momentum preservation and asymmetric ridge traversal provides a baseline for future refinement.

Nonparametric statistical analyses corroborate the empirical performance observations. According to the Friedman ranking (presented in Table 2), DBRCCO achieves the primary rank with an average score of 1.5172, outperforming DE and the baseline CCO. The Wilcoxon signed-rank test *p*-values reported in Table 3 fall below the 0.05 significance threshold across most comparisons. This statistical superiority confirms that the optimization gains achieved by DBRCCO represent an architectural advancement rather than numerical variance.

### 4.4. Ablation Study

To demonstrate the independent contributions of the Dung Beetle local search (DBO) and the Reflecting Boundary Handling Strategy (RBHS), we conducted an ablation study using the CEC2017 benchmark suite. Four algorithm variants were compared: the original CCO framework, a CCO variant augmented with the local search strategy (DBO-CCO), a CCO variant equipped with the reflecting boundary mechanism (Ref-CCO), and the integrated architecture (DBRCCO). The results are summarized in Table 4.

The results in Table 4 demonstrate that the combination of the DBO strategy and RBHS enhances search performance. This combination allows the algorithm to escape local optima more effectively than either strategy in isolation, achieving the highest number of “Best” evaluations and the primary Friedman ranking.

The ablation data highlights the superiority of the Reflecting Boundary Handling Strategy (RBHS) over the standard clamping technique employed by the baseline CCO. The standard clamping mechanism is defined as

(15) $${\left(x_{i}^{j}\right)}^{\mathrm{clamp}}=\left\{\begin{array}{cc} u^{j}, & \mathrm{if}\;x_{i}^{j}>u^{j}\\ l^{j}, & \mathrm{if}\;x_{i}^{j}<l^{j}\\ x_{i}^{j}, & \mathrm{otherwise} \end{array}\right.$$

where ${\left(x_{i}^{j}\right)}^{\mathrm{clamp}}$ represents the position component after enforcing the limitation. While clamping forces out-of-bounds values onto the perimeter limits ($u^{j}$ or $l^{j}$), it destroys the momentum of the search vector. In contrast, the reflecting mechanism acts as a perfectly elastic wall. By retaining the velocity magnitude and reversing its directional vector back into the feasible space, RBHS prevents population diversity loss at domain edges. As validated by Ref-CCO’s improved statistical variance over CCO in Table 4, this momentum-preserving trajectory is vital for maintaining continuous exploration and accelerating convergence.

## 5. Application to Production Optimization

Reservoir production optimization is a challenge in petroleum engineering aimed at operational strategies that maximize the economic value of field development, quantified by the Net Present Value (NPV). The objective is to dynamically modulate the injection and extraction rates of subsurface fluids over time to maximize profitability. This physical system exhibits complex fluid dynamics across numerous wells over multiple time steps, classifying it as an NP-hard optimization problem. Consequently, gradient-free metaheuristic algorithms are well-suited for this task, given their capability to traverse non-convex search spaces without derivative evaluations.

To assess its practical utility, the proposed DBRCCO framework is applied within a reservoir simulation workflow. The Eclipse simulator is used to model a synthetic multi-channel reservoir scenario. The algorithm iteratively proposes control configurations for production and injection wells across management intervals. The simulator evaluates each proposed configuration, outputting fluid production behavior and economic viability.

The objective is to maximize the NPV, formulated as

(16) $$NPV\left(x,z\right)=\sum_{t=1}^{n}\frac{\Delta t\cdot (Q_{o,t}\cdot r_{o}-Q_{w,t}\cdot r_{w}-Q_{i,t}\cdot r_{i})}{{\left(1+b\right)}^{p_{t}}}$$

Here, x and z denote the control variable vector and reservoir state vector, respectively; $Q_{o,t}$, $Q_{w,t}$, and $Q_{i,t}$ represent the oil production, water production, and water injection rates during time step *t*; $r_{o}$, $r_{w}$, and $r_{i}$ are the associated unit price and cost factors; *b* is the annual discount rate; and $p_{t}$ is the time period corresponding to the cash flow. Nonlinear production constraints are omitted, simplifying the problem to an unconstrained maximization.

### 5.1. Reservoir Model Description

**Figure 3:**
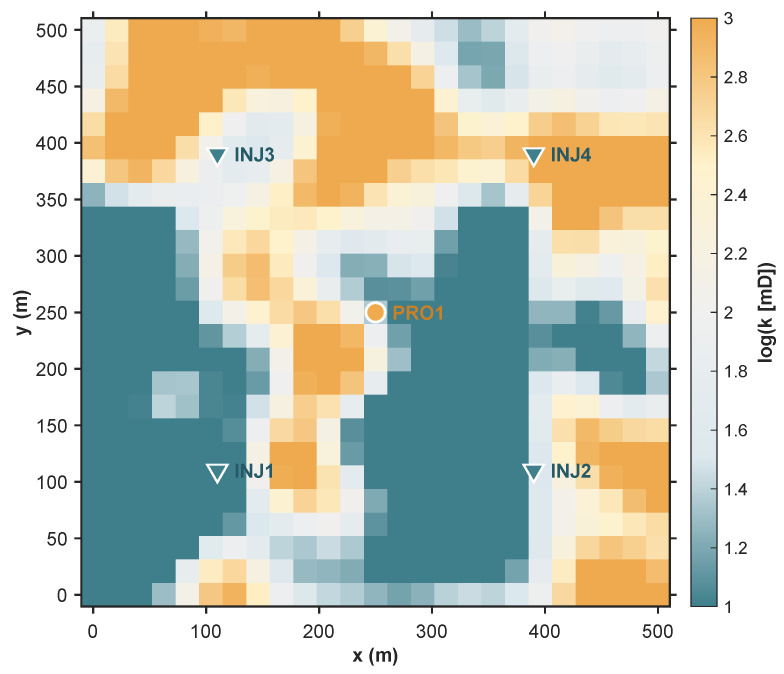
Two-dimensional synthetic reservoir model and well pattern arrangement.

A two-dimensional synthetic reservoir model simulates flow characteristics in a fluvial depositional environment. It employs a standard five-spot well pattern featuring a central production well (PRO1) and four corner injection wells (INJ1 through INJ4), as depicted in Figure 3. This configuration provides a framework for assessing the optimization methodology under subsurface heterogeneity.

The geological domain is spatially discretized using a uniform Cartesian grid of 25×25 cells, yielding exactly 625 active simulation blocks. Each grid cell has a uniform thickness and planar dimensions of 20×20 meters. A constant porosity of 0.20 is enforced throughout the model. The permeability field is stochastically generated to simulate the spatial variability of fluvial environments, featuring high-permeability sinuous channels embedded within a low-permeability background matrix. The resulting log-permeability (ln(K)) map, governing primary fluid migration pathways, is presented in Figure 3.

The production lifetime spans 1000 to 2000 days, subdivided into 5 to 15 discrete control intervals. Optimization on four or five wells yields a problem with 20 to 75 decision variables. The objective is to maximize Net Present Value (NPV) by determining optimal well controls per interval. Economic parameters, including oil price (100 USD/STB), water injection and processing costs (8 USD/STB each), and an annual discount rate (3%), are assigned plausible values. This ensures economic realism while focusing on core algorithmic performance.

### 5.2. Analysis and Discussion of Experimental Results

This subsection evaluates DBRCCO against eight comparative metaheuristic algorithms applied to the reservoir production optimization problem. To ensure statistical integrity, each algorithm was independently executed five times under identical hardware and software conditions. The primary evaluative metrics include the mean net present value (Mean NPV), standard deviation (Std), and the peak and minimum values recorded across all executions.

The outcomes detailed in Table 5 demonstrate the consistent performance of DBRCCO.

DBRCCO achieves the highest mean NPV of USD $9.954\times 10^{8}$ and the lowest standard deviation of USD $0.852\times 10^{7}$. This translates to a 12.54% economic improvement relative to the baseline CCO algorithm. Assuming a normal distribution of the independent runs, the calculated 95% Confidence Interval (CI) for DBRCCO’s mean performance spans between $\left[9.879\times 10^{8},1.0029\times 10^{9}\right]$. This narrow confidence bound, situated above the peak values of competing models (such as DE and MGO in Table 5), indicates reproducible optimization without vulnerability to sample deviations. Among the competitors, Differential Evolution (DE) and Moss Growth Optimization (MGO) deliver the next-best mean results, though with standard deviations two to three times larger than DBRCCO’s. Algorithms such as the Parrot Optimizer (PO), Slime Mould Algorithm (SMA), and Bat Algorithm (BA) yield the lowest mean NPVs and exhibit the highest standard deviations.

**Figure 4:**
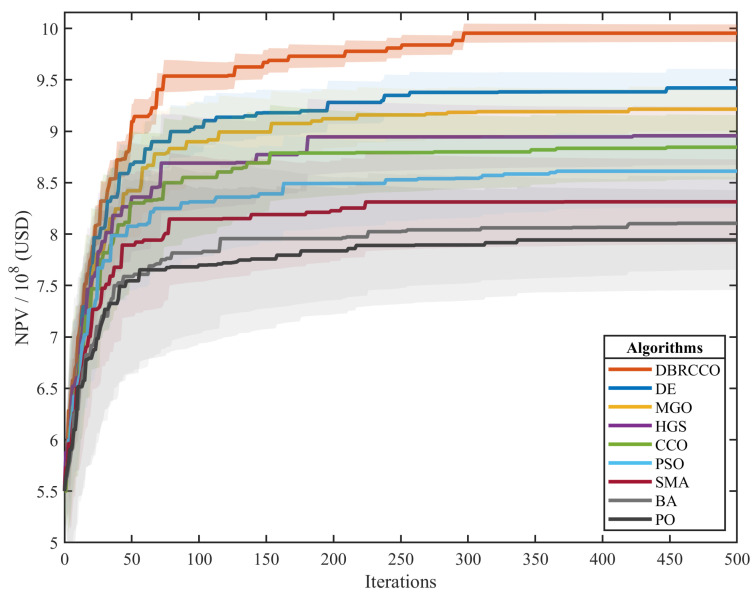
Convergence curve analysis of all algorithms on the production optimization problem.

The convergence behavior of all algorithms is further illustrated in Figure 4. Figure 4 graphically depicts the progression of the Net Present Value (NPV) over iterations for each algorithm across a representative run of the production optimization experiment, with the x-axis representing computational iterations and the y-axis the objective function value (NPV in USD).

The convergence trajectories substantiate the statistical rankings in Table 5. DBRCCO exhibits an efficient search profile, characterized by a rapid, monotonic ascent toward the highest recorded NPV plateau. Its curve is smooth, indicating steady improvement without stagnation or oscillations. Conversely, while DE and MGO demonstrate competitive convergence velocities, their resultant curves exhibit markedly slower initial ascents and slightly more pronounced variance before stabilizing at lower final economic values. In contrast, algorithms with lower mean performance, particularly PO, SMA, and BA, show significantly slower convergence, with trajectories that plateau early at suboptimal values or exhibit considerable volatility, reflecting a poor exploration–exploitation balance.

These trends correspond to the quantitative results: the steep, stable ascent of DBRCCO signifies its capacity for both exploration and exploitation, leading to high-quality solutions. The convergence behavior corroborates the algorithm’s performance and robustness evidenced in Table 5.

In summary, DBRCCO achieves the highest economic outcome and stable convergence for the reservoir production optimization problem. Its performance, characterized by the highest mean NPV and lowest standard deviation, confirms the efficacy of its enhanced exploration–exploitation balance and search mechanisms. These findings validate the algorithm’s practical applicability in solving complex engineering optimization problems, providing a foundation for its deployment in real-field scenarios.

**Figure 5:**
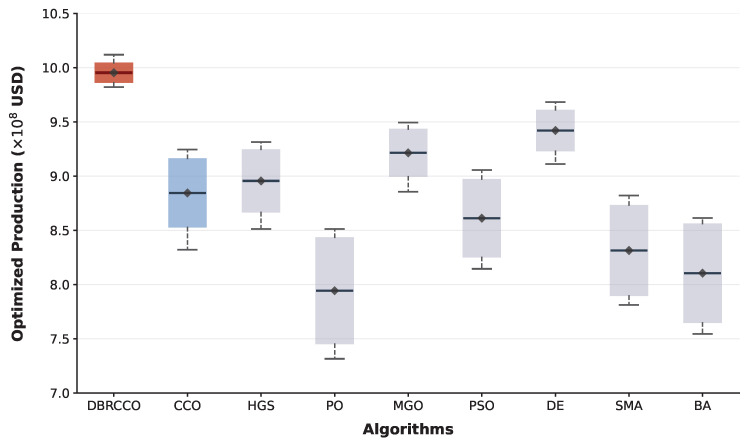
Boxplot of the Net Present Value (NPV) obtained by different algorithms on the production optimization problem.

Figure 5 presents a boxplot distribution constructed from the extreme values and central tendencies detailed in Table 5. DBRCCO separates itself from the competitors, occupying the highest profitability quadrant. The compactness of its box and the high lower-bound (Worst) value corroborate its superior resistance to stagnation. In contrast, while DE and MGO exhibit high peak performances, their extended whiskers and broader interquartile ranges indicate significant variance across independent runs. Algorithms such as PO and SMA exhibit downward skewness, failing to exploit the high-yield regions of the reservoir model. These distributional characteristics affirm DBRCCO’s capabilities as a robust optimizer for computationally expensive engineering tasks.

## 6. Conclusions

This study introduced the Dung Beetle with Reflection Cuckoo Catfish Optimizer (DBRCCO), a metaheuristic algorithm formulated to resolve the limitations inherent within the CCO architecture. By integrating an adaptive, dung beetle-inspired localized search mechanism and a momentum-preserving reflecting boundary strategy, DBRCCO rectifies CCO’s deficiency in fine-grained exploitation and inefficient boundary constraints.

Empirical evaluations across 29 CEC2017 benchmark functions demonstrated DBRCCO’s statistical performance. When benchmarked against eight metaheuristic algorithms, DBRCCO achieved a global Friedman ranking score of 1.5172 and demonstrated superiority across the Wilcoxon signed-rank tests (p<0.05). Furthermore, in solving a reservoir production optimization problem, DBRCCO outperformed competing methodologies. It achieved a Mean Net Present Value (NPV) of USD $9.954\times 10^{8}$, representing a 12.54% economic improvement over the original CCO framework. Moreover, DBRCCO exhibited structural robustness, constraining the standard deviation to merely USD $0.852\times 10^{7}$, equivalent to a 72.7% reduction in outcome variance relative to the baseline algorithm. These successes validate the practical efficacy of DBRCCO.

Future work will focus on extending the applicability of DBRCCO to high-dimensional optimization scenarios. Research directions include its adaptation for constrained runs and multi-objective formulations. Furthermore, investigations into the parameter sensitivity of the integrated mechanisms, complemented by their application to domain-specific engineering challenges, are planned to ascertain the algorithmic versatility of the proposed methodology.

## Figures and Tables

**Table 1:** CEC2017 benchmark functions.

Function	Function Name	Class	Optimum
F1	Shifted and Rotated Bent Cigar Function	Unimodal	100
F3	Shifted and Rotated Zakharov Function	Unimodal	300
F4	Shifted and Rotated Rosenbrock’s Function	Multimodal	400
F5	Shifted and Rotated Rastrigin’s Function	Multimodal	500
F6	Shifted and Rotated Expanded Scaffer’s F6 Function	Multimodal	600
F7	Shifted and Rotated Lunacek Bi-Rastrigin Function	Multimodal	700
F8	Shifted and Rotated Non-Continuous Rastrigin’s Function	Multimodal	800
F9	Shifted and Rotated Lévy Function	Multimodal	900
F10	Shifted and Rotated Schwefel’s Function	Multimodal	1000
F11	Hybrid Function 1 (*n* = 3)	Hybrid	1100
F12	Hybrid Function 2 (*n* = 3)	Hybrid	1200
F13	Hybrid Function 3 (*n* = 3)	Hybrid	1300
F14	Hybrid Function 4 (*n* = 4)	Hybrid	1400
F15	Hybrid Function 5 (*n* = 4)	Hybrid	1500
F16	Hybrid Function 6 (*n* = 4)	Hybrid	1600
F17	Hybrid Function 6 (*n* = 5)	Hybrid	1700
F18	Hybrid Function 6 (*n* = 5)	Hybrid	1800
F19	Hybrid Function 6 (*n* = 5)	Hybrid	1900
F20	Hybrid Function 6 (*n* = 6)	Hybrid	2000
F21	Composition Function 1 (*n* = 3)	Composition	2100
F22	Composition Function 2 (*n* = 3)	Composition	2200
F23	Composition Function 3 (*n* = 4)	Composition	2300
F24	Composition Function 4 (*n* = 4)	Composition	2400
F25	Composition Function 5 (*n* = 5)	Composition	2500
F26	Composition Function 6 (*n* = 5)	Composition	2600
F27	Composition Function 7 (*n* = 6)	Composition	2700
F28	Composition Function 8 (*n* = 6)	Composition	2800
F29	Composition Function 9 (*n* = 3)	Composition	2900
F30	Composition Function 10 (*n* = 3)	Composition	3000

**Table 2:** Results of the DBRCCO and Other Algorithms on CEC2017 Benchmark Functions. Bold values indicate the best (minimum) mean result for each function.

	F1		F3		F4	
**Algo.**	**Avg**	**Std**	**Avg**	**Std**	**Avg**	**Std**
DBRCCO	$1.0000\times 10^{2}$	$3.4688\times 10^{-14}$	$3.0015\times 10^{2}$	$3.2352\times 10^{-1}$	$4.1210\times 10^{2}$	$2.3570\times 10^{1}$
CCO	$4.8995\times 10^{3}$	$4.9682\times 10^{3}$	$3.0001\times 10^{2}$	$8.1379\times 10^{-3}$	$5.1453\times 10^{2}$	$2.6365\times 10^{1}$
HGS	$8.0232\times 10^{3}$	$6.6301\times 10^{3}$	$1.4131\times 10^{3}$	$3.3199\times 10^{3}$	$4.8121\times 10^{2}$	$2.1374\times 10^{1}$
PO	$6.4116\times 10^{7}$	$1.1976\times 10^{8}$	$4.9564\times 10^{3}$	$2.9905\times 10^{3}$	$5.2696\times 10^{2}$	$2.1539\times 10^{1}$
MGO	$8.2239\times 10^{4}$	$5.3668\times 10^{4}$	$4.3657\times 10^{4}$	$1.1034\times 10^{4}$	$4.9681\times 10^{2}$	$1.2642\times 10^{1}$
PSO	$2.0170\times 10^{3}$	$2.7236\times 10^{3}$	$3.0000\times 10^{2}$	$6.1013\times 10^{-3}$	$4.5355\times 10^{2}$	$3.1647\times 10^{1}$
DE	$1.0046\times 10^{3}$	$1.3850\times 10^{3}$	$1.9329\times 10^{4}$	$5.7523\times 10^{3}$	$4.9011\times 10^{2}$	$8.2413\times 10^{0}$
SMA	$2.3637\times 10^{9}$	$9.8913\times 10^{8}$	$4.0229\times 10^{4}$	$9.6663\times 10^{3}$	$6.4889\times 10^{2}$	$8.3808\times 10^{1}$
BA	$5.6764\times 10^{5}$	$3.7942\times 10^{5}$	$3.0012\times 10^{2}$	$1.1344\times 10^{-1}$	$4.7712\times 10^{2}$	$3.7121\times 10^{1}$
	**F5**		**F6**		**F7**	
**Algo.**	**Avg**	**Std**	**Avg**	**Std**	**Avg**	**Std**
DBRCCO	$5.6610\times 10^{2}$	$1.5513\times 10^{1}$	$6.0000\times 10^{2}$	$4.3310\times 10^{-4}$	$7.8910\times 10^{2}$	$9.8031\times 10^{0}$
CCO	$7.0369\times 10^{2}$	$2.9049\times 10^{1}$	$6.4965\times 10^{2}$	$4.9194\times 10^{0}$	$1.0364\times 10^{3}$	$8.4640\times 10^{1}$
HGS	$6.3295\times 10^{2}$	$3.5939\times 10^{1}$	$6.0147\times 10^{2}$	$1.2875\times 10^{0}$	$8.7917\times 10^{2}$	$5.0027\times 10^{1}$
PO	$7.0861\times 10^{2}$	$4.0838\times 10^{1}$	$6.5499\times 10^{2}$	$6.8901\times 10^{0}$	$1.1242\times 10^{3}$	$7.0424\times 10^{1}$
MGO	$5.6632\times 10^{2}$	$1.2655\times 10^{1}$	$6.0000\times 10^{2}$	$3.3762\times 10^{-5}$	$7.9950\times 10^{2}$	$1.3307\times 10^{1}$
PSO	$6.9433\times 10^{2}$	$2.7818\times 10^{1}$	$6.4466\times 10^{2}$	$8.0225\times 10^{0}$	$1.0253\times 10^{3}$	$7.3166\times 10^{1}$
DE	$6.0720\times 10^{2}$	$8.7311\times 10^{0}$	$6.0000\times 10^{2}$	$2.3206\times 10^{-14}$	$8.4644\times 10^{2}$	$7.2669\times 10^{0}$
SMA	$7.1472\times 10^{2}$	$2.6860\times 10^{1}$	$6.4233\times 10^{2}$	$8.8345\times 10^{0}$	$1.0755\times 10^{3}$	$6.1452\times 10^{1}$
BA	$8.2094\times 10^{2}$	$5.9430\times 10^{1}$	$6.7273\times 10^{2}$	$1.0345\times 10^{1}$	$1.6469\times 10^{3}$	$1.4731\times 10^{2}$
	**F8**		**F9**		**F10**	
**Algo.**	**Avg**	**Std**	**Avg**	**Std**	**Avg**	**Std**
DBRCCO	$8.6272\times 10^{2}$	$1.4967\times 10^{1}$	$9.8491\times 10^{2}$	$7.5246\times 10^{1}$	$3.0720\times 10^{3}$	$2.6138\times 10^{2}$
CCO	$9.4395\times 10^{2}$	$1.8624\times 10^{1}$	$3.2873\times 10^{3}$	$7.5277\times 10^{2}$	$5.1585\times 10^{3}$	$5.9521\times 10^{2}$
HGS	$9.1782\times 10^{2}$	$2.3606\times 10^{1}$	$3.5338\times 10^{3}$	$9.7411\times 10^{2}$	$3.8941\times 10^{3}$	$5.2169\times 10^{2}$
PO	$9.7070\times 10^{2}$	$2.9049\times 10^{1}$	$5.3667\times 10^{3}$	$8.4863\times 10^{2}$	$5.5128\times 10^{3}$	$7.3098\times 10^{2}$
MGO	$8.6751\times 10^{2}$	$9.8904\times 10^{0}$	$9.4902\times 10^{2}$	$4.5041\times 10^{1}$	$4.7532\times 10^{3}$	$3.2898\times 10^{2}$
PSO	$9.4614\times 10^{2}$	$3.6992\times 10^{1}$	$4.2248\times 10^{3}$	$9.2261\times 10^{2}$	$5.3123\times 10^{3}$	$5.4460\times 10^{2}$
DE	$9.1180\times 10^{2}$	$6.6621\times 10^{0}$	$9.0000\times 10^{2}$	$4.6412\times 10^{-14}$	$5.8423\times 10^{3}$	$2.6826\times 10^{2}$
SMA	$9.6287\times 10^{2}$	$2.5893\times 10^{1}$	$5.8105\times 10^{3}$	$7.2556\times 10^{2}$	$5.8664\times 10^{3}$	$6.1171\times 10^{2}$
BA	$1.0688\times 10^{3}$	$7.6061\times 10^{1}$	$1.3980\times 10^{4}$	$4.2622\times 10^{3}$	$5.8367\times 10^{3}$	$5.4241\times 10^{2}$
	**F11**		**F12**		**F13**	
**Algo.**	**Avg**	**Std**	**Avg**	**Std**	**Avg**	**Std**
DBRCCO	$1.1290\times 10^{3}$	$1.6514\times 10^{1}$	$7.1767\times 10^{3}$	$9.4079\times 10^{3}$	$1.3447\times 10^{3}$	$1.2759\times 10^{1}$
CCO	$1.2601\times 10^{3}$	$5.5552\times 10^{1}$	$2.9118\times 10^{6}$	$2.5568\times 10^{6}$	$2.5179\times 10^{4}$	$1.4497\times 10^{4}$
HGS	$1.2069\times 10^{3}$	$3.6797\times 10^{1}$	$7.9104\times 10^{5}$	$6.5701\times 10^{5}$	$3.0371\times 10^{4}$	$2.6414\times 10^{4}$
PO	$1.2993\times 10^{3}$	$4.7668\times 10^{1}$	$2.8152\times 10^{7}$	$2.5306\times 10^{7}$	$9.9752\times 10^{4}$	$7.1972\times 10^{4}$
MGO	$1.1868\times 10^{3}$	$2.4761\times 10^{1}$	$1.3325\times 10^{6}$	$1.0072\times 10^{6}$	$2.5409\times 10^{4}$	$2.2080\times 10^{4}$
PSO	$1.2087\times 10^{3}$	$2.3490\times 10^{1}$	$3.4133\times 10^{4}$	$1.7472\times 10^{4}$	$1.2954\times 10^{4}$	$1.2026\times 10^{4}$
DE	$1.1594\times 10^{3}$	$2.4084\times 10^{1}$	$1.7444\times 10^{6}$	$7.9114\times 10^{5}$	$3.0543\times 10^{4}$	$1.4428\times 10^{4}$
SMA	$1.5521\times 10^{3}$	$9.4235\times 10^{1}$	$9.1860\times 10^{7}$	$3.4659\times 10^{7}$	$2.0463\times 10^{6}$	$2.0540\times 10^{6}$
BA	$1.2941\times 10^{3}$	$5.8046\times 10^{1}$	$2.3036\times 10^{6}$	$1.8418\times 10^{6}$	$3.1912\times 10^{5}$	$1.3021\times 10^{5}$
	**F14**		**F15**		**F16**	
**Algo.**	**Avg**	**Std**	**Avg**	**Std**	**Avg**	**Std**
DBRCCO	$1.4303\times 10^{3}$	$1.3894\times 10^{1}$	$1.5185\times 10^{3}$	$8.8780\times 10^{0}$	$2.1987\times 10^{3}$	$1.8378\times 10^{2}$
CCO	$1.6443\times 10^{3}$	$5.2795\times 10^{1}$	$9.4325\times 10^{3}$	$4.4089\times 10^{3}$	$2.7431\times 10^{3}$	$2.6284\times 10^{2}$
HGS	$3.3332\times 10^{4}$	$2.6758\times 10^{4}$	$1.8847\times 10^{4}$	$1.4362\times 10^{4}$	$2.7338\times 10^{3}$	$2.6983\times 10^{2}$
PO	$3.8566\times 10^{4}$	$2.7042\times 10^{4}$	$6.2698\times 10^{4}$	$4.4467\times 10^{4}$	$3.1222\times 10^{3}$	$3.5402\times 10^{2}$
MGO	$1.3350\times 10^{4}$	$1.1490\times 10^{4}$	$1.7599\times 10^{4}$	$1.4845\times 10^{4}$	$2.2082\times 10^{3}$	$1.1379\times 10^{2}$
PSO	$6.2066\times 10^{3}$	$2.8402\times 10^{3}$	$7.0953\times 10^{3}$	$8.5587\times 10^{3}$	$2.8880\times 10^{3}$	$3.3030\times 10^{2}$
DE	$5.6518\times 10^{4}$	$2.6020\times 10^{4}$	$8.0268\times 10^{3}$	$5.1455\times 10^{3}$	$2.0954\times 10^{3}$	$1.2337\times 10^{2}$
SMA	$1.7217\times 10^{5}$	$1.1024\times 10^{5}$	$1.8131\times 10^{4}$	$8.7162\times 10^{3}$	$2.8418\times 10^{3}$	$2.9756\times 10^{2}$
BA	$5.8470\times 10^{3}$	$3.7413\times 10^{3}$	$9.9844\times 10^{4}$	$3.9482\times 10^{4}$	$3.5078\times 10^{3}$	$4.5963\times 10^{2}$
	**F17**		**F18**		**F19**	
**Algo.**	**Avg**	**Std**	**Avg**	**Std**	**Avg**	**Std**
DBRCCO	$1.8623\times 10^{3}$	$9.9550\times 10^{1}$	$1.8685\times 10^{3}$	$1.1721\times 10^{2}$	$1.9147\times 10^{3}$	$5.0879\times 10^{0}$
CCO	$2.2523\times 10^{3}$	$2.0401\times 10^{2}$	$2.1947\times 10^{4}$	$6.8637\times 10^{3}$	$4.5975\times 10^{3}$	$3.9299\times 10^{3}$
HGS	$2.2649\times 10^{3}$	$2.0790\times 10^{2}$	$3.6351\times 10^{5}$	$3.0179\times 10^{5}$	$2.8358\times 10^{4}$	$2.3690\times 10^{4}$
PO	$2.2922\times 10^{3}$	$1.9807\times 10^{2}$	$5.4028\times 10^{5}$	$4.7240\times 10^{5}$	$8.0836\times 10^{5}$	$7.7336\times 10^{5}$
MGO	$1.8765\times 10^{3}$	$5.0568\times 10^{1}$	$3.4402\times 10^{5}$	$1.8792\times 10^{5}$	$1.1756\times 10^{4}$	$9.3131\times 10^{3}$
PSO	$2.4815\times 10^{3}$	$2.8086\times 10^{2}$	$1.7859\times 10^{5}$	$1.6106\times 10^{5}$	$7.8586\times 10^{3}$	$7.1298\times 10^{3}$
DE	$1.8427\times 10^{3}$	$5.5387\times 10^{1}$	$2.9694\times 10^{5}$	$1.1593\times 10^{5}$	$6.9108\times 10^{3}$	$3.5551\times 10^{3}$
SMA	$2.3065\times 10^{3}$	$2.1386\times 10^{2}$	$4.8288\times 10^{5}$	$4.0199\times 10^{5}$	$3.9400\times 10^{5}$	$5.0933\times 10^{5}$
BA	$2.7562\times 10^{3}$	$3.6191\times 10^{2}$	$2.4196\times 10^{5}$	$1.8138\times 10^{5}$	$5.7814\times 10^{5}$	$2.2356\times 10^{5}$
	**F20**		**F21**		**F22**	
**Algo.**	**Avg**	**Std**	**Avg**	**Std**	**Avg**	**Std**
DBRCCO	$2.1781\times 10^{3}$	$1.1141\times 10^{2}$	$2.3609\times 10^{3}$	$3.7296\times 10^{1}$	$2.7240\times 10^{3}$	$1.0082\times 10^{3}$
CCO	$2.5194\times 10^{3}$	$1.4940\times 10^{2}$	$2.4831\times 10^{3}$	$3.8738\times 10^{1}$	$2.3015\times 10^{3}$	$1.8283\times 10^{0}$
HGS	$2.4802\times 10^{3}$	$1.8467\times 10^{2}$	$2.4203\times 10^{3}$	$3.7820\times 10^{1}$	$5.3462\times 10^{3}$	$1.0168\times 10^{3}$
PO	$2.5365\times 10^{3}$	$1.7030\times 10^{2}$	$2.5035\times 10^{3}$	$4.6569\times 10^{1}$	$3.5944\times 10^{3}$	$1.9613\times 10^{3}$
MGO	$2.2455\times 10^{3}$	$7.5763\times 10^{1}$	$2.3676\times 10^{3}$	$2.6196\times 10^{1}$	$3.1542\times 10^{3}$	$1.5331\times 10^{3}$
PSO	$2.6783\times 10^{3}$	$1.8494\times 10^{2}$	$2.4940\times 10^{3}$	$5.6463\times 10^{1}$	$6.0369\times 10^{3}$	$1.7673\times 10^{3}$
DE	$2.1473\times 10^{3}$	$6.7714\times 10^{1}$	$2.4116\times 10^{3}$	$9.2045\times 10^{0}$	$3.9859\times 10^{3}$	$1.8185\times 10^{3}$
SMA	$2.4266\times 10^{3}$	$1.3715\times 10^{2}$	$2.4673\times 10^{3}$	$2.4224\times 10^{1}$	$4.0818\times 10^{3}$	$2.1244\times 10^{3}$
BA	$2.9744\times 10^{3}$	$3.1257\times 10^{2}$	$2.6512\times 10^{3}$	$8.5552\times 10^{1}$	$7.2326\times 10^{3}$	$1.3280\times 10^{3}$
	**F23**		**F24**		**F25**	
**Algo.**	**Avg**	**Std**	**Avg**	**Std**	**Avg**	**Std**
DBRCCO	$2.7171\times 10^{3}$	$2.1884\times 10^{1}$	$2.9310\times 10^{3}$	$3.1475\times 10^{1}$	$2.8872\times 10^{3}$	$2.2434\times 10^{0}$
CCO	$3.0781\times 10^{3}$	$1.0002\times 10^{2}$	$3.1941\times 10^{3}$	$1.1192\times 10^{2}$	$2.9294\times 10^{3}$	$2.7886\times 10^{1}$
HGS	$2.7635\times 10^{3}$	$2.9589\times 10^{1}$	$3.0080\times 10^{3}$	$5.5451\times 10^{1}$	$2.8892\times 10^{3}$	$9.0922\times 10^{0}$
PO	$2.9351\times 10^{3}$	$6.6121\times 10^{1}$	$3.1040\times 10^{3}$	$7.5358\times 10^{1}$	$2.9308\times 10^{3}$	$2.1991\times 10^{1}$
MGO	$2.7175\times 10^{3}$	$1.3834\times 10^{1}$	$2.8839\times 10^{3}$	$4.9904\times 10^{1}$	$2.8876\times 10^{3}$	$1.1187\times 10^{0}$
PSO	$3.3415\times 10^{3}$	$1.6042\times 10^{2}$	$3.3141\times 10^{3}$	$8.9628\times 10^{1}$	$2.8817\times 10^{3}$	$5.9179\times 10^{0}$
DE	$2.7584\times 10^{3}$	$8.2395\times 10^{0}$	$2.9550\times 10^{3}$	$1.0057\times 10^{1}$	$2.8874\times 10^{3}$	$3.1412\times 10^{-1}$
SMA	$2.8673\times 10^{3}$	$3.7060\times 10^{1}$	$3.0089\times 10^{3}$	$3.0073\times 10^{1}$	$2.9915\times 10^{3}$	$3.7808\times 10^{1}$
BA	$3.3451\times 10^{3}$	$1.4790\times 10^{2}$	$3.3765\times 10^{3}$	$1.4108\times 10^{2}$	$2.9123\times 10^{3}$	$2.2845\times 10^{1}$
	**F26**		**F27**		**F28**	
**Algo.**	**Avg**	**Std**	**Avg**	**Std**	**Avg**	**Std**
DBRCCO	$3.6005\times 10^{3}$	$7.7061\times 10^{2}$	$3.2118\times 10^{3}$	$9.6251\times 10^{0}$	$3.1365\times 10^{3}$	$5.5311\times 10^{1}$
CCO	$4.3791\times 10^{3}$	$2.1554\times 10^{3}$	$3.5583\times 10^{3}$	$1.5197\times 10^{2}$	$3.2203\times 10^{3}$	$3.8572\times 10^{1}$
HGS	$4.9662\times 10^{3}$	$5.8146\times 10^{2}$	$3.2297\times 10^{3}$	$1.8408\times 10^{1}$	$3.2106\times 10^{3}$	$4.3833\times 10^{1}$
PO	$6.9140\times 10^{3}$	$1.2377\times 10^{3}$	$3.2938\times 10^{3}$	$4.9522\times 10^{1}$	$3.3114\times 10^{3}$	$3.9078\times 10^{1}$
MGO	$4.1193\times 10^{3}$	$4.0424\times 10^{2}$	$3.2125\times 10^{3}$	$6.6128\times 10^{0}$	$3.2307\times 10^{3}$	$1.7519\times 10^{1}$
PSO	$6.9583\times 10^{3}$	$1.8915\times 10^{3}$	$3.2512\times 10^{3}$	$2.0492\times 10^{2}$	$3.1522\times 10^{3}$	$5.1535\times 10^{1}$
DE	$4.6266\times 10^{3}$	$1.1596\times 10^{2}$	$3.2057\times 10^{3}$	$3.1582\times 10^{0}$	$3.1930\times 10^{3}$	$4.3502\times 10^{1}$
SMA	$5.2223\times 10^{3}$	$6.1833\times 10^{2}$	$3.2535\times 10^{3}$	$2.2070\times 10^{1}$	$3.4089\times 10^{3}$	$3.9210\times 10^{1}$
BA	$9.2598\times 10^{3}$	$1.6717\times 10^{3}$	$3.4728\times 10^{3}$	$1.5937\times 10^{2}$	$3.1259\times 10^{3}$	$5.3921\times 10^{1}$
	**F29**		**F30**			
**Algo.**	**Avg**	**Std**	**Avg**	**Std**		
DBRCCO	$3.4316\times 10^{3}$	$8.8275\times 10^{1}$	$5.1060\times 10^{3}$	$1.2901\times 10^{2}$		
CCO	$4.4248\times 10^{3}$	$3.3299\times 10^{2}$	$2.5317\times 10^{5}$	$2.9750\times 10^{5}$		
HGS	$3.7884\times 10^{3}$	$1.9716\times 10^{2}$	$9.6813\times 10^{4}$	$1.1734\times 10^{5}$		
PO	$4.4290\times 10^{3}$	$3.6004\times 10^{2}$	$8.3528\times 10^{6}$	$5.9620\times 10^{6}$		
MGO	$3.6311\times 10^{3}$	$8.6782\times 10^{1}$	$5.6112\times 10^{4}$	$3.7957\times 10^{4}$		
PSO	$4.0101\times 10^{3}$	$3.4655\times 10^{2}$	$5.5220\times 10^{3}$	$2.2222\times 10^{3}$		
DE	$3.5143\times 10^{3}$	$7.4482\times 10^{1}$	$1.2904\times 10^{4}$	$3.5403\times 10^{3}$		
SMA	$4.0658\times 10^{3}$	$1.9904\times 10^{2}$	$5.5422\times 10^{6}$	$4.6386\times 10^{6}$		
BA	$4.9408\times 10^{3}$	$3.8035\times 10^{2}$	$1.4166\times 10^{6}$	$6.7815\times 10^{5}$		
**Overall Rank**						
**Algo.**			**RANK**	**+/=/−**	**AVG Rank**	**Avg Time (s)**
DBRCCO			1	∼	1.5172	21.4
CCO			6	26/2/1	5.0	20.5
HGS			4	28/1/0	4.6897	25.6
PO			9	29/0/0	7.3793	31.2
MGO			3	19/9/1	3.6552	28.9
PSO			5	25/2/2	4.7931	18.5
DE			2	23/2/4	3.4138	19.2
SMA			7	29/0/0	7.2069	34.5
BA			8	27/2/0	7.3448	14.5

**Table 3:** The *p*-values of the DBRCCO versus other algorithms on CEC2017.

Fun	CCO	HGS	PO	MGO	PSO	DE	SMA	BA
F1	$1.23\times 10^{-5}$	$1.23\times 10^{-5}$	$1.23\times 10^{-5}$	$1.23\times 10^{-5}$	$1.23\times 10^{-5}$	$1.23\times 10^{-5}$	$1.23\times 10^{-5}$	$1.23\times 10^{-5}$
F3	$6.65\times 10^{-4}$	$1.23\times 10^{-5}$	$1.23\times 10^{-5}$	$1.23\times 10^{-5}$	$3.62\times 10^{-5}$	$1.23\times 10^{-5}$	$1.23\times 10^{-5}$	$6.19\times 10^{-1}$
F4	$1.23\times 10^{-5}$	$1.23\times 10^{-5}$	$1.23\times 10^{-5}$	$1.39\times 10^{-5}$	$7.22\times 10^{-5}$	$1.23\times 10^{-5}$	$1.23\times 10^{-5}$	$1.23\times 10^{-5}$
F5	$1.23\times 10^{-5}$	$1.23\times 10^{-5}$	$1.23\times 10^{-5}$	$8.61\times 10^{-1}$	$1.23\times 10^{-5}$	$1.23\times 10^{-5}$	$1.23\times 10^{-5}$	$1.23\times 10^{-5}$
F6	$1.23\times 10^{-5}$	$1.23\times 10^{-5}$	$1.23\times 10^{-5}$	$9.80\times 10^{-2}$	$1.23\times 10^{-5}$	$1.23\times 10^{-5}$	$1.23\times 10^{-5}$	$1.23\times 10^{-5}$
F7	$1.23\times 10^{-5}$	$1.23\times 10^{-5}$	$1.23\times 10^{-5}$	$5.36\times 10^{-3}$	$1.23\times 10^{-5}$	$1.23\times 10^{-5}$	$1.23\times 10^{-5}$	$1.23\times 10^{-5}$
F8	$1.23\times 10^{-5}$	$1.23\times 10^{-5}$	$1.23\times 10^{-5}$	$2.31\times 10^{-1}$	$1.23\times 10^{-5}$	$1.23\times 10^{-5}$	$1.23\times 10^{-5}$	$1.23\times 10^{-5}$
F9	$1.23\times 10^{-5}$	$1.23\times 10^{-5}$	$1.23\times 10^{-5}$	$1.09\times 10^{-1}$	$1.23\times 10^{-5}$	$1.23\times 10^{-5}$	$1.23\times 10^{-5}$	$1.23\times 10^{-5}$
F10	$1.23\times 10^{-5}$	$3.62\times 10^{-5}$	$1.23\times 10^{-5}$	$1.23\times 10^{-5}$	$1.23\times 10^{-5}$	$1.23\times 10^{-5}$	$1.23\times 10^{-5}$	$1.23\times 10^{-5}$
F11	$1.23\times 10^{-5}$	$1.39\times 10^{-5}$	$1.23\times 10^{-5}$	$2.26\times 10^{-5}$	$1.23\times 10^{-5}$	$1.26\times 10^{-4}$	$1.23\times 10^{-5}$	$1.23\times 10^{-5}$
F12	$1.23\times 10^{-5}$	$1.23\times 10^{-5}$	$1.23\times 10^{-5}$	$1.23\times 10^{-5}$	$2.86\times 10^{-5}$	$1.23\times 10^{-5}$	$1.23\times 10^{-5}$	$1.23\times 10^{-5}$
F13	$1.23\times 10^{-5}$	$1.23\times 10^{-5}$	$1.23\times 10^{-5}$	$1.23\times 10^{-5}$	$1.23\times 10^{-5}$	$1.23\times 10^{-5}$	$1.23\times 10^{-5}$	$1.23\times 10^{-5}$
F14	$1.23\times 10^{-5}$	$1.23\times 10^{-5}$	$1.23\times 10^{-5}$	$1.23\times 10^{-5}$	$1.23\times 10^{-5}$	$1.23\times 10^{-5}$	$1.23\times 10^{-5}$	$1.23\times 10^{-5}$
F15	$1.23\times 10^{-5}$	$1.23\times 10^{-5}$	$1.23\times 10^{-5}$	$1.23\times 10^{-5}$	$1.23\times 10^{-5}$	$1.23\times 10^{-5}$	$1.23\times 10^{-5}$	$1.23\times 10^{-5}$
F16	$2.00\times 10^{-5}$	$1.77\times 10^{-5}$	$1.39\times 10^{-5}$	$8.19\times 10^{-1}$	$1.77\times 10^{-5}$	$3.47\times 10^{-2}$	$2.00\times 10^{-5}$	$1.23\times 10^{-5}$
F17	$1.77\times 10^{-5}$	$2.00\times 10^{-5}$	$1.77\times 10^{-5}$	$7.37\times 10^{-1}$	$1.23\times 10^{-5}$	$4.43\times 10^{-1}$	$1.23\times 10^{-5}$	$1.23\times 10^{-5}$
F18	$1.23\times 10^{-5}$	$1.23\times 10^{-5}$	$1.23\times 10^{-5}$	$1.23\times 10^{-5}$	$1.23\times 10^{-5}$	$1.23\times 10^{-5}$	$1.23\times 10^{-5}$	$1.23\times 10^{-5}$
F19	$1.23\times 10^{-5}$	$1.23\times 10^{-5}$	$1.23\times 10^{-5}$	$1.23\times 10^{-5}$	$1.23\times 10^{-5}$	$1.23\times 10^{-5}$	$1.23\times 10^{-5}$	$1.23\times 10^{-5}$
F20	$1.23\times 10^{-5}$	$5.13\times 10^{-5}$	$1.77\times 10^{-5}$	$1.49\times 10^{-2}$	$1.23\times 10^{-5}$	$3.00\times 10^{-1}$	$2.86\times 10^{-5}$	$1.23\times 10^{-5}$
F21	$1.23\times 10^{-5}$	$5.76\times 10^{-5}$	$1.23\times 10^{-5}$	$4.43\times 10^{-1}$	$1.23\times 10^{-5}$	$1.39\times 10^{-5}$	$1.23\times 10^{-5}$	$1.23\times 10^{-5}$
F22	$1.35\times 10^{-1}$	$1.77\times 10^{-5}$	$4.53\times 10^{-3}$	$4.93\times 10^{-3}$	$3.22\times 10^{-5}$	$6.85\times 10^{-3}$	$6.85\times 10^{-3}$	$1.23\times 10^{-5}$
F23	$1.23\times 10^{-5}$	$1.26\times 10^{-4}$	$1.23\times 10^{-5}$	$7.78\times 10^{-1}$	$1.23\times 10^{-5}$	$1.39\times 10^{-5}$	$1.23\times 10^{-5}$	$1.23\times 10^{-5}$
F24	$1.39\times 10^{-5}$	$2.26\times 10^{-5}$	$1.39\times 10^{-5}$	$3.62\times 10^{-5}$	$1.23\times 10^{-5}$	$9.80\times 10^{-4}$	$1.39\times 10^{-5}$	$1.23\times 10^{-5}$
F25	$1.57\times 10^{-5}$	$6.96\times 10^{-1}$	$1.39\times 10^{-5}$	$2.70\times 10^{-3}$	$2.40\times 10^{-4}$	$7.42\times 10^{-3}$	$1.23\times 10^{-5}$	$1.13\times 10^{-4}$
F26	$4.59\times 10^{-1}$	$6.45\times 10^{-5}$	$1.23\times 10^{-5}$	$2.14\times 10^{-2}$	$4.07\times 10^{-5}$	$2.26\times 10^{-5}$	$3.22\times 10^{-5}$	$1.23\times 10^{-5}$
F27	$1.23\times 10^{-5}$	$4.03\times 10^{-4}$	$1.23\times 10^{-5}$	$5.27\times 10^{-1}$	$1.49\times 10^{-2}$	$3.82\times 10^{-3}$	$1.39\times 10^{-5}$	$1.23\times 10^{-5}$
F28	$3.22\times 10^{-5}$	$1.74\times 10^{-4}$	$1.23\times 10^{-5}$	$1.39\times 10^{-5}$	$1.79\times 10^{-1}$	$1.19\times 10^{-3}$	$1.23\times 10^{-5}$	$4.12\times 10^{-1}$
F29	$1.23\times 10^{-5}$	$1.57\times 10^{-5}$	$1.23\times 10^{-5}$	$2.00\times 10^{-5}$	$1.39\times 10^{-5}$	$5.82\times 10^{-3}$	$1.23\times 10^{-5}$	$1.23\times 10^{-5}$
F30	$1.23\times 10^{-5}$	$1.23\times 10^{-5}$	$1.23\times 10^{-5}$	$1.23\times 10^{-5}$	$9.46\times 10^{-1}$	$1.23\times 10^{-5}$	$1.23\times 10^{-5}$	$1.23\times 10^{-5}$

**Table 4:** Ablation study results for DBRCCO on CEC 2017. “Best” signifies the number of functions where the variant outperformed others. “Mean Rank” denotes the average Friedman ranking. The “+/≈/−” column summarizes the Wilcoxon rank-sum test results (α=0.05) relative to DBRCCO. The most favorable results are highlighted in bold. The gray-shaded row highlights the proposed method.

Algorithm	Best	Mean Rank	+/≈/−
**DBRCCO (Proposed)**	**24**	**1.21**	—
DBO-CCO	4	2.15	4/6/19
Ref-CCO	1	3.02	0/7/22
CCO (Original)	0	3.62	0/3/26

**Table 5:** Experimental Results of All Algorithms on the Production Optimization Problem.

Algorithm	Mean (USD)	Std	Best (USD)	Worst (USD)
DBRCCO	$9.954\times 10^{8}$	$0.852\times 10^{7}$	$1.012\times 10^{9}$	$9.821\times 10^{8}$
CCO	$8.845\times 10^{8}$	$3.125\times 10^{7}$	$9.245\times 10^{8}$	$8.321\times 10^{8}$
HGS	$8.956\times 10^{8}$	$2.841\times 10^{7}$	$9.314\times 10^{8}$	$8.512\times 10^{8}$
PO	$7.943\times 10^{8}$	$4.856\times 10^{7}$	$8.512\times 10^{8}$	$7.315\times 10^{8}$
MGO	$9.215\times 10^{8}$	$2.134\times 10^{7}$	$9.495\times 10^{8}$	$8.856\times 10^{8}$
PSO	$8.612\times 10^{8}$	$3.542\times 10^{7}$	$9.056\times 10^{8}$	$8.145\times 10^{8}$
DE	$9.421\times 10^{8}$	$1.845\times 10^{7}$	$9.684\times 10^{8}$	$9.112\times 10^{8}$
SMA	$8.314\times 10^{8}$	$4.125\times 10^{7}$	$8.821\times 10^{8}$	$7.812\times 10^{8}$
BA	$8.105\times 10^{8}$	$4.512\times 10^{7}$	$8.614\times 10^{8}$	$7.545\times 10^{8}$

## Data Availability

The numerical and experimental data used to support the findings of this study are included within the article.

## Abbreviations

The following abbreviations are used in this manuscript:

DBRCCO	Dung Beetle with Reflection Cuckoo Catfish Optimizer
CCO	Cuckoo Catfish Optimizer
NPV	Net Present Value
BHP	Bottom-Hole Pressure
RPO	Reservoir Production Optimization
CEC	Congress on Evolutionary Computation
EA	Evolutionary Algorithm
SI	Swarm Intelligence
GA	Genetic Algorithm
DE	Differential Evolution
PSO	Particle Swarm Optimization
ACO	Ant Colony Optimization
NFL	No Free Lunch
